# Beyond interpretation: relational value of shared words in multilingual psychiatry

**DOI:** 10.1192/bjb.2026.10228

**Published:** 2026-06

**Authors:** Kamran Mahmood

**Affiliations:** ST6 General Adult Psychiatry, Bradford District Care NHS Foundation Trust, Bradford, UK

Language lies at the heart of psychiatry. Our work depends on words, their meaning and context. Yet, in increasingly multicultural UK mental health settings, especially where I work, psychiatrists and patients often do not share a mother tongue. Professional interpreters are essential for accuracy and safety, but the emotional and symbolic power of language can be overlooked. In this reflection, I explore how even a few words in a patient’s own language, simple greetings or everyday phrases, can deepen rapport and transform therapeutic engagement. Two encounters taught me that communication in psychiatry extends far beyond literal meaning.

The first was with a middle-aged Persian man with post-traumatic stress disorder, referred for cognitive–behavioural therapy with behavioural activation. In our early sessions, he was visibly anxious: tense posture, little eye contact, minimal speech and frequent sips of water. I do not speak Persian, but my mother tongue, Urdu, shares some cultural resonances. At the start of our second session, I welcomed him in Persian with ‘khush-aamaddeed’. His expression softened instantly; he smiled, and something shifted. Sensing the value of that moment, I added a brief weekly ritual: he would teach me one Persian word. What began as an icebreaker became a therapeutic tool. He arrived prepared, chose the word carefully and took pride in teaching. This small act appeared to enhance his sense of agency, shifting him from passive patient to active participant. Over the weeks he became more relaxed, more engaged in behavioural tasks, and increasingly at ease, sometimes laughing when I mispronounced a word. Here, language became a bridge to trust, embodying respect, curiosity and validation of identity. It reminded me that recovery depends not only on evidence-based interventions but also on the quality of human connection.

The second patient, a young Kurdish man speaking Turkish, presented with adjustment disorder. When I saw him in the waiting room with head down, closed posture, I greeted him in Turkish: ‘Hosh Geldiniz’. His face lit up. While waiting for an interpreter, I asked, ‘Nasılsınız?’ – meaning how are you. He responded with a smile. I had learned those words decades earlier at a Turkish school in Pakistan. Over two meetings, those simple phrases softened his demeanour each time. When the telephone interpreter repeatedly failed owing to poor reception, I used Google Translate, showing him typed questions and reading his responses. To my surprise, he became more expressive and energetic. Though not a substitute for professional interpretation, this direct exchange revealed something important: communication is not only about information but about feeling seen and heard.

These encounters highlighted the essence of transcultural psychiatric practice. Greeting a patient in their language is not merely linguistic, it is relational. It conveys effort, humility and openness, qualities universally understood. As Kleinman notes, the clinician in cross-cultural work often becomes the learner. That role reversal can foster empowerment and mutual respect.

These moments reminded me why I entered psychiatry. Amid protocols and pressures, I felt simply human. A few words in another’s tongue can carry profound empathy. Psychiatry, at its core, is about being human together.

